# Bioactivities and Health Benefits of Wild Fruits

**DOI:** 10.3390/ijms17081258

**Published:** 2016-08-04

**Authors:** Ya Li, Jiao-Jiao Zhang, Dong-Ping Xu, Tong Zhou, Yue Zhou, Sha Li, Hua-Bin Li

**Affiliations:** 1 Guangdong Provincial Key Laboratory of Food, Nutrition and Health, School of Public Health, Sun Yat-Sen University, Guangzhou 510080, China; liya28@mail2.sysu.edu.cn (Y.L.); zhangjj46@mail2.sysu.edu.cn (J.-J.Z.); xudp@mail2.sysu.edu.cn (D.-P.X.); zhout43@mail2.sysu.edu.cn (T.Z.); zhouyue3@mail2.sysu.edu.cn (Y.Z.); 2 School of Chinese Medicine, The University of Hong Kong, Hong Kong, China; u3003781@connect.hku.hk; 3 South China Sea Bioresource Exploitation and Utilization Collaborative Innovation Center, Sun Yat-Sen University, Guangzhou 510006, China

**Keywords:** wild fruit, bioactivity, antioxidant, anticancer, anti-inflammatory

## Abstract

Wild fruits are exotic or underutilized. Wild fruits contain many bioactive compounds, such as anthocyanins and flavonoids. Many studies have shown that wild fruits possess various bioactivities and health benefits, such as free radical scavenging, antioxidant, anti-inflammatory, antimicrobial, and anticancer activity. Therefore, wild fruits have the potential to be developed into functional foods or pharmaceuticals to prevent and treat several chronic diseases. In the present article, we review current knowledge about the bioactivities and health benefits of wild fruits, which is valuable for the exploitation and utilization of wild fruits.

## 1. Introduction

Fruits and vegetables, containing abundant dietary fiber, vitamins, and minerals, in particular large amounts of phytochemicals [1,2,3,4,5,6,7], are recommended by nutritionists because of their health benefits [8,9]. Phytochemicals in these natural products are considered to be responsible for positive health outcomes. Particularly, it is widely noted that plants produce a great deal of antioxidants to combat the oxidative stress induced by oxygen and light in the natural environment [10]. Oxidative stress performs an essential role in multiple chronic diseases [11,12,13]. Therefore, antioxidants in fruit and vegetables have been extensively explored for their effects on several diseases. Epidemiological and nutritional studies suggested that the higher one’s fruit and vegetable consumption, the lower the incidence of chronic diseases such as coronary heart problems, cancer, and Alzheimer’s disease [14,15].

Wild fruits are fruits of wild plants, and are often exotic, underutilized, or less known. Many wild fruits are safe to consume, and some have been developed as medicines. Due to different genotypes and environmental concerns, wild fruits contain rich phytochemicals such as anthocyanin and flavonoids. Therefore, wild fruits are often considered to be healthy foods. In recent years, wild fruits have attracted increasing attention, and accumulative investigations have been performed for their bioactive effects, such as antioxidant, antimicrobial anti-inflammatory, and anticancer effects. These studies pointed out that wild fruits could have the potential to prevent and treat some chronic diseases. This review summarizes the bioactivities and health benefits of wild fruits.

## 2. Bioactivities of Wild Fruits

### 2.1. Antioxidant Activity

Free radicals are normally produced as a byproduct of cellular metabolism. Free radicals are capable of killing bacteria, damaging biomolecules, provoking immune responses, activating oncogenes, causing atherogenesis, and enhancing the ageing process [16]. The most important classes of radical species generated in living systems are reactive oxygen and nitrogen species (ROS and RNS). The excessive production of ROS and RNS could play a pivotal part in many human chronic diseases, including atherosclerosis, diabetes mellitus, cancer, rheumatoid arthritis, cataract, and Parkinson’s disease [17]. Various natural products have been proved to have antioxidant activities, such as fruits, vegetables, edible flowers, cereal grains, wine, herbal plants, and their tea infusions [18,19,20,21,22,23,24,25,26]. Therefore, natural resources of antioxidants have been considered as quite important. There have been several experiments both in vivo and in vitro proving that many wild fruits possess antioxidant activities, such as wild blueberries, wild apples, and wild hawthorn fruits.

#### 2.1.1. In Vitro Studies

Several studies have evaluated the antioxidant capacity of a certain species of wild fruit. The underutilized wild berry fruit *Prunus mahaleb* showed strong antioxidant activity [27]. The results of oxygen radical absorption capacity (ORAC) and 2,2′-azinobis-3-ethylbenzothiazoline-6-sulphonate (ABTS·^+^, expressed as trolox equivalent antioxidant capacity (TEAC) value) assays were 150 and 45 mmol Trolox equivalents/kg fresh weight, respectively. Furthermore, the *P. mahaleb* fruit had high anthocyanin content, which was comparable to that of some reported superfruits (bilberries and blackcurrants). Moreover, Araca-pera (*Psidium acutangulum*), an exotic guava fruit from the Amazon, was analyzed for antioxidant properties by 2,2-diphenyl-1-picrylhydrazyl (DPPH) free radical, ABTS free radical scavenging capacity (24.96 ± 0.75, 90.57 ± 0.63 mg of vitamin C/100 g fresh fruit, respectively), and cell-based assays (76%–100%) [28]. Results indicated that this guava fruit could be developed into functional foods for the prevention of chronic diseases due to its antioxidant activity. In another study, the antioxidant activities of water, ethyl acetate, acetone, and methanol extracts from the wild *Sorbus torminalis* fruit were assessed by DPPH, ABTS, superoxide anion radicals scavenging, and ferric reducing antioxidant power (FRAP) assays. The antioxidant activity and total phenolic concentration were both ranked as water > ethyl acetate > acetone > methanol extracts [29]. Another edible wild fruit, *Ziziphus mistol*, was analyzed for its antioxidant activity. All extracts showed strong antioxidant activity. As a hydrogen or electron donor, the ethanol extract (EME) was significantly more effective than the aqueous one (AME); when scavenging hydroxyl and superoxide radicals, AME was significantly more effective than EME. In addition, a dose-dependent relationship (*R*^2^ > 0.90) was found between polyphenols content and antioxidant capacity. These results suggested that consumption of *Ziziphus mistol* fruit could be encouraged due to its antioxidant activity [30]. The pulp of wild cherimoya fruits (*Annona cherimola*) was also assessed for the antioxidant capacities of its methanol, ethanol, and dimethyl formamide extracts. The three extracts all showed strong free radical capturing and antioxidant activities. Among them, the dimethyl formamide extract showed the highest DPPH and ABTS scavenging and FRAP activities, and the ethanol extract showed the strongest anti-lipid peroxidation activity [31]. In another study, a crude extract of *Myrica esculenta* fruit was assessed for antioxidant properties. Results showed that the extracts exhibited considerable antioxidant potential based on data from DPPH, ABTS, and FRAP assays. Moreover, the antioxidant capacity was positively correlated with total phenolic and total flavonoids contents [32]. The wild bilberry (*Vaccinium meridionale*) is an edible fruit from Colombia. Garzon et al. evaluated its antioxidant activity and the results of ABTS and FRAP assays proved its strong antioxidant activity [33]. In another study, the antioxidant activity of fruit from wild *Lycium ruthenicum*, a nutritional food that has been used in traditional Chinese medicine, was evaluated. The methanol extracts exhibited high antioxidant activity in ABTS, DPPH, and FRAP assays [34]. In addition, hydrophilic extracts of wild acerola (*Malpighia emarginata*) pulps and juices were analyzed for antioxidant activities. Results of DPPH, ABTS, and FRAP assays indicated that the antioxidant activity of acerola juice was stronger than that of the fruit juices reported in the literature, such as strawberry, grape, or apple. In addition, anthocyanins, flavonoids, and phenolic acids fractions were separated; among them, phenolic acids showed the highest antioxidant activity, indicating that phenolic acids contributed the most to the antioxidant property of wild acerola fruit [35]. Furthermore, Koca et al. analyzed the antioxidant activity of purple mulberry (*Morus rubra*) fruits growing wild in Turkey. FRAP assay was used and the average value was 33.90 μmol/g [36]. In addition, fruits of wild *Bunium persicum*, *Elaeagnus latifolia*, *Solanum incanum*, *Rosa canina*, *Mespilus germanica*, *Aristotelia chilensis*, *Myrtus communis*, *Rubus hirsutus*, *Piper capense*, *Vitis coignetiae*, *Prunus spinosa*, *Syzygium cumini*, and *Vatis amurensis* also showed strong antioxidant activities [37,38,39,40,41,42,43,44,45,46,47,48,49]; the related information is displayed in Table 1.

Some studies compared the antioxidant activities between different genotypes of a certain species of wild fruit. Fourteen wild mandarin genotypes of *Citrus reticulata* were assessed for the antioxidant activities and phenolic compounds in the peels [50]. Results showed that antioxidant potency composite (APC) index varied from 58.84 to 98.89 in the studied wild genotypes, and among them, Nieduyeju showed the highest APC index. Furthermore, wild genotypes Guangxihongpisuanju, Nieduyeju, Cupigoushigan, and Daoxianyeju contained more phenolic compounds and exhibited higher antioxidant capacities than the commercial cultivars Satsuma and Ponkan. In another study, ethanol and ethyl acetate extracts of 10 crabapple varieties (*Malus* wild species) from China were analyzed for the antioxidant activities. Ethyl acetate extract showed higher contents of total phenolic and total flavonoids, and stronger DPPH and ABTS radical scavenging activities than ethanol extract, while ethanol extract had a significantly higher FRAP value (*p* < 0.01) than ethyl acetate extract. Results also showed that whole fruits of wild *Malus* species, particularly *Malus rockii*, exhibited stronger antioxidant activity than reported apple peel, indicating that *Malus* wild species could be rich sources of antioxidants [51]. Papaya, a fruit of the genus *Chaenomeles*, is an important source of functional food and traditional Chinese herbs. Du et al. evaluated the total polyphenol content (TPC) and antioxidant potential of five wild *Chaenomeles* genotypes [52]. Among them, the fruit of *C. speciosa* showed the highest free radical scavenging abilities by ABTS and FRAP assays while the *C. thibetica* extract was less effective. *C. sinensis* showed the highest DPPH scavenging capacity. Among them, DPPH values of extracts from four genotypes, *C. sinensis* (6.48 ± 0.23), *C. speciosa* (5.63 ± 0.17), *C. thibetica* (4.89 ± 0.21), and *C. cathayensis* (4.88 ± 0.25), were higher than Trolox (3.79 ± 0.07). In addition, wild genotypes of *Vaccinium* berries were evaluated for their differences in bioactivity on oxidative protection and minimum dosage to have a significant action [53]. Wild *Vaccinium* extracts are 3.04-fold more active than cultivated extracts by EC_50_, indicating that wild *Vaccinium* berries possessed stronger antioxidant activity than the cultivated ones. The results of six antioxidant assays showed a good relationship with anthocyanin and polyphenol content. In addition, the essential oil (EO) compositions and antioxidant activities of wild fruits *Hypericum perforatum* and *Hypericum scabrum* were analyzed. It was found that the antioxidant abilities of the EOs evaluated by β-carotene bleaching and DPPH assays might be due to their α-pinene contents [54]. Furthermore, the total phenolics and antioxidant activity of a group of *Fragaria* genotypes were determined and compared with the commercial genotype *F. xananassa*. The antioxidant capacity in the wild material was about three-fold higher than the commercial material [55]. In another study, wild bananas (*Ensete superbum*) had higher contents of phenolics and tannins, higher DPPH, ABTS, and FRAP activities than commercial ones [56]. Furthermore, all the investigated wild strawberry accessions (*Fragaria vesca*) showed higher antioxidant activity than the commercial cultivar (Camarosa) [57]. Results of another study indicated a significant difference between different wild strawberry fruits in their abilities to scavenge DPPH radicals [58]. Furthermore, a wild strawberry showed higher total phenolics and antioxidant activity than those cultivated samples [59]. Two wild raspberries also showed high antioxidant activity by FRAP, ABTS, and DPPH assays [60]. Besides, six genotypes of *Diospyros kaki* fruits were analyzed, and wild genotype *D. kaki* var. Silvestris Makino showed the highest content of phenolics and strongest antioxidant activity [61]. Additionally, eight wild genotypes of *Rosa canina* fruit showed great antioxidant activity, with a good relationship with total polyphenols and vitamin C content [62]. Several genotypes of wild bitter gourd (*Momordica charantia*) from Taiwan showed protective activity against Cu^2+^-induced low-density-lipoprotein peroxidation [63]. Furthermore, fruits of 10 wild almonds (*Prunus amygdalus*) were assessed, and two kinds (*A. pabotti* Browicz and *A. orientalis* Duhamel) exhibited the best antioxidant properties [64]. In addition, four wild almond species from Iran showed strong antioxidant activity [67]. Moreover, two wild blueberries showed higher total polyphenols content and antioxidant activity than three cultivated ones [65]. In addition, the fruit of the wild lime (*Citrus hystrix*) had higher antioxidant, flavonoid, and phenolic contents than cultivated ones [66]. In another study, wild blueberry exhibited stronger antioxidant activity than four cultivated ones [68]. Two wild berries showed potent antioxidant activity by ORAC assay [69]. However, in another study, the antioxidant capacities of fruits of wild and cultivated cranberries were similar, without a statistically significant difference (*p* < 0.05) [70]. Furthermore, wild cranberries exhibited a lower average antioxidant capacity than cultivated berries [71]. Similarly, hydroalcoholic extracts of wild murtilla (*Ugni molinae*) fruit showed weaker DPPH· and ABTS· scavenging capacity than cultivated ones [72].

Some studies screened different species of wild fruits for their antioxidant activities. In a study, 12 native Australian fruits were screened for the antioxidant activities and contents of phenolics, anthocyanins, and ascorbic acid, using ABTS and photochemiluminescence (PCL) assays [73]. Among them, five fruits exhibited significant stronger radical scavenging abilities (3.1- to 5.2-fold and 1.2- to 4.2-fold for ABTS and PCL assays, respectively) than blueberry (used as control). Six studied fruits showed higher total phenolics content (2.5- to 3.9-fold of control). Moreover, the Kakadu plum had the highest content of ascorbic acid (938-fold of control). These fruits could be a novel rich source of natural antioxidants. In another study, Fu et al. evaluated the antioxidant abilities of 56 exotic fruits from south China. Results of FRAP and ABTS·^+^ (expressed as TEAC value) assays showed that these fruits generally possessed high antioxidant capacities, which were strongly correlated with total phenolic content, indicating that phenolic compounds mainly contributed to their antioxidant activities [3]. In another study, 14 wild fruits were assessed for their antioxidant activities [74]. Results showed that among all the tested fruits, the acetone extract of *Detarium microcarpum* fruit possessed the highest DPPH free radical scavenging capacity, FRAP values, and ABTS free radical scavenging capacity. Meanwhile, antioxidant activities were strongly correlated with total phenolic and flavonoid levels. In addition, antioxidant activity was evaluated for seed residue extracts of wild *Rubus ulmifolius* and *Sambucus nigra* fruits [75]. The results of a DPPH assay showed significant antioxidant capacities of the extracts from all fruit seed residues. Meanwhile, the methanolic extract of *Rubus* seed residue exhibited a stronger antioxidant activity than that of *Sambucus* seed. In another study, Malta et al. tested the antioxidant activities of three wild cerrado fruits called gabiroba (*Campomanesia cambessedeana*), murici (*Byrsonoma verbascifolia*), and guapeva (*Pouteria guardneriana*) [76]. Results showed that gabiroba fruit was the richest source of total phenolics, and exhibited the highest antioxidant activity for both assays (ORAC, peroxyl radical scavenging capacity assays). In addition, ethanol extracts of three wild fruits, genipap (*Genipa americana*), umbu (*Spondia tuberosa*), and siriguela (*Spondia purpurea*), were analyzed for their antioxidant capacities. Siriguela and umbu (seeds and peels) extracts exhibited the highest antioxidant activities. Results of lipid peroxidation assay showed that pulp of genipap could be a promising source of antioxidant [77]. Furthermore, three wild fruits, *Rubus megalococcus*, *Myrciaria aft cauliflora*, and *Hyeronima macrocarpa*, were tested for their antioxidant activities. Results showed that the anthocyanin-rich extracts of *Hyeronima macrocarpa* exhibited stronger radical scavenging activity than the other extracts [78]. Moreover, 11 fresh exotic fruits from Brazil were analyzed for antioxidant activities by DPPH and ABTS assays. All the fruits showed considerable antioxidant activity, and the phenolic contents were positively correlated with total antioxidant activity by ABTS (*R* = 0.94, *p* ≤ 0.001) and DPPH (*R* = 0.88, *p* ≤ 0.001) assays [79]. In another study, 15 wild fruits were screened for their antioxidant activities [80]. Results showed that fruits of wild *Terminalia bellirica*, *Terminalia chebula*, *Phyllanthus emblica*, and *Spondias pinnata* possessed the strongest antioxidant activity based on the DPPH assay. Moreover, *Spondias pinnata* was more effective (16% radical scavenging activity) than vitamin C (5% radical scavenging activity), both at 5 μg/mL. Additionally, the peel and pulp of six wild fruits, sour plum (*Ximenia caffra*), marula (*Sclerocarya birrea*), mobola plum (*Parinari curatellifolia*), chocolate berry (*Vitex payos*), velvet sweet-berry (*Bridelia molis*), and red ivory (*Berchemia zeyheri*), were tested for their antioxidant activities. Both the peel and pulp of sour plum showed higher reducing capacities than all the other fruits, while velvet sweet-berry, the peel and pulp of sour plum, and chocolate berry peel showed high inhibitory effects on phospholipid peroxidation at high concentrations [81]. Additionally, the exotic Camu-camu fruit (*Myrciaria dubia*) presented the highest DPPH·scavenging capacity of all the fruits tested [82]. Furthermore, several wild blackberry fruit samples showed strong antioxidant activity with rich phenolic profile and content [83]. In another study, results showed the order of the antioxidant activity of five wild fruits was *Rhus semialata* > *Docynia indica* > *Garcinia xanthochymus* > *Averrhoa carambola* > *Garcinia pedunculata* [84]. In addition, wild *Arbustus unedo* fruit showed higher Folin–Ciocalteu values, vitamin C, and phenolic content than *Rubus ulmifolius* fruit [85]. Wild blackthorn (*Prunus spinose*) fruit exhibited higher antioxidant capacity than hawthorn (*Crataegus monogyna*) fruit [86]. In addition, several exotic tropical fruits (bacuri, caja, camu-camu, carnauba, gurguri, jabuticaba, jambolao, jucara, murta, black puca, and puca) showed strong antioxidant activity in a DPPH assay [87]. Additionally, fruit of wild *Rosa canina* showed higher efficacy towards ABTS· and H_2_O_2_ species than other tested wild fruits [88]. Moreover, methanolic extracts from jackal berry (*Diospyros mespiliformis*) showed higher DPPH radical scavenging capacity compared with other tested fruits [89]. In addition, fruits of *Fragaria indica*, *Prunus armeniaca*, *Pyracantha crenulata*, and *Rubus ellipticus* showed strong antioxidant activity [90]. Furthermore, 20 exotic fruits showed high antioxidant activity [91]. In addition, 24 exotic fruits were assessed, and the highest antioxidant activity and content of total phenolics were observed in banana passion fruits (*Passiflora tarminiana* and *Passiflora mollisima*) [92]. Similarly, exotic acerola showed the highest antioxidant values in the 10 exotic fruits investigated [93], and exotic dovialis showed the strongest antioxidant activity among the investigated exotic fruits [94]. In addition, wild *Psidium cattleianum*, *Averrhoa carambola*, *Syzygium cumini*, and *Psidium guajava* fruits showed the highest antioxidant capacities among the 17 exotic fruits from Mauritius [95]. Furthermore, polyphenolic extracts of three wild red berry fruits (*Cornus mas*, *Prunus spinose*, and *Rubus fruticosus*) showed strong scavenging ability on DPPH radical (IC_50_ values of 22.19 to 31.18 mL/g) [96].

#### 2.1.2. In Vivo Studies

Several studies also evaluated the antioxidant activities of some wild fruits in vivo. In a study, wild snake fruit (*Salacca edulis*) and mangosteen (*Garcinia mangostana*) were analyzed for their influences on antioxidant activities and plasma lipids in rats fed with cholesterol. The rats were fed with diets supplemented with snake fruit and mangosteen for four weeks, and it was found that the increase in plasma lipids and the decline in antioxidant activity were both hindered, and snake fruit was more effective than mangosteen [97]. In another study, the effect of a polyphenol-rich extract (PrB) of *Vaccinium angustifolium* (wild blueberries) on brain oxidative status in adult, male, 3–4-month-old Balb-c mice was examined. Antioxidant status was determined by FRAP assay and levels of ascorbic acid, malondialdehyde, and reduced glutathione in whole brain homogenates. Lipid peroxidation products were decreased (38% and 79%) and brain ascorbic acid level was increased (21% and 64%) in both PrB30- and PrB60-treated groups. An increased glutathione level (28%) was observed in the PrB60-treated group. The results indicated that the fruit possessed strong brain antioxidant property [98]. In addition, the in vivo antioxidant activities of rare exotic Thai fruits, durian, snake fruit, and mangosteen, were investigated. Results showed that plasma lipid profile and antioxidant activity in rats fed with cholesterol-containing diets were positively influenced by diets supplemented with these exotic fruits [99].

These studies proved that abundant wild fruits could be potential sources of natural antioxidants, thus supporting their full utilization as bioactive elements in the food, pharmaceutical, and cosmetic industries. The antioxidant activity and possible functional components of extracts of some wild fruits are summarized in Table 1.

### 2.2. Antimicrobial Activity

It is well known that various bacterial, fungal, and viral species could cause plant, animal, and human diseases, thereby causing the loss of crops, food spoilage, or even food poisoning that could damage human health [100,101]. Hence, it is important to develop natural effective antimicrobial agents. In recent years, wild fruits have exhibited potential antibacterial, antifungal, and antiviral activities in several studies.

#### 2.2.1. Antibacterial and Antifungal Activities

Some studies analyzed the antimicrobial activity of a certain species of wild fruit. An aqueous extract of wild fruit *Nitraria retusa* was tested for inhibition of microbial growth in beef patties. The results showed that the extract possessed strong antimicrobial activity against *Salmonella typhimurium*, *Klebsiella pneumonia*, and *Bacillus thuringiensis* [102]. In addition, extracts of wild yellow azarole fruit peel showed considerable antibacterial activity, especially against *Staphylococcus aureus* and *Streptococcus faecalis* [103]. Moreover, methanol and n-hexane extracts from fruits of wild mahaleb cherry (*Prunus mahaleb*) were screened by measuring their inhibitory activity on several bacteria (*Escherichia coli*, *Pseudomonas aeruginosa*, *Proteus mirabilis*, *K. pneumoniae*, *Acinetobacter baumannii*, *S. aureus*, *Enterococcus faecalis*, and *Bacillus subtilis*), as well as several fungi (*Candida albicans*, *Candida parapsilosis*, *Candida tropicalis*, and *Candida krusei*). The extracts showed antibacterial activity against both Gram (+) bacteria and Gram (−) bacteria tested, and the methanol and n-hexane extracts showed antifungal activity against *C. krusei* [104]. Moreover, a fresh fruit extract of the wild plant *Clematis apiifolia* exhibited minimum inhibitory concentrations (MIC) in the vicinity of 0.1% against various yeasts and non-lactic acid bacteria of ≤0.4%. MICs against lactic acid bacteria were about 2.0%. Results indicated that this fruit was even more effective in antibacterial activity than garlic, which has great antibacterial properties. Furthermore, the principal antimicrobial compound of *C. apiifolia* was isolated and identified as protoanemonin. The researchers suggested that the antimicrobial compound of *C. apiifolia* inhibited microorganisms by reacting with sulfhydryl groups of cellular proteins [105]. Furthermore, the antimicrobial activities of the essential oil hydrodistilled from wild pepper fruits were evaluated. Results showed medium inhibitory effect against the Gram (+) species *E. faecalis*, *S. aureus*, and the yeast *C. albicans* [59]. Moreover, inhibitory effects on the growth of *Mycobacterium tuberculosis* H(37)Rv was observed in fruits of wild ampalaya (*Momordica charantia*). The fruits of wild ampalaya showed higher antitubercular activity (90%) than that of the cultivated variety (81%) [106]. Malek et al. tested the antibacterial activities of oils separated from the fruit of *Scabiosa arenaria*, a wild plant growing in Tunisia. The 16 Gram (+) and Gram (−) bacteria and four *Candida* species were used. The oils exhibited significant inhibitory activities against these bacterial and *Candida* species, superior to thymol, which was used as a positive control [107].

*Carissa opaca* is a wild plant used widely in ethnomedicine. Thirty-four strains of Gram (+) and Gram (−) bacteria were used to determine the antibacterial activities of ethanol extracts of the fruits. The results exhibited a broad spectrum of efficacy [108]. Additionally, crude oils from ripe and unripe wild olive fruits were proved to have antibacterial activity against some of the Gram (+) and Gram (−) bacterial strains [109]. In another study, antimicrobial properties of extracts of fruits from wild melon (*Citrullus lanatus*) were tested. The researchers tested antimicrobial properties of crude chloroform, hexane, and ethanol extracts against five bacteria (*E. coli*, *S. aureus*, *P. aeruginosa*, *B. subtilis*, and *Proteus vulgaris*) and two fungi (*Aspergillus nigar* and *C. albican*). It was found that a chloroform extract of the fruit showed the highest antibacterial activity, while an ethanol extract of the fruit pulp exhibited the highest antifungal activity. It is worth mentioning that the fruit of this plant was as potent as standard antimicrobial drugs (clotrimazole and gentamici) against certain microorganisms [110]. Moreover, another study showed that wild strawberry guavas (*Psidium cattleianum*) possessed better antimicrobial activity than common guavas [111]. In addition, different wild clones of European cranberry were investigated for their antimicrobial activities. Results showed that extracts of wild European cranberry had inhibitory effects against the growth of varieties of human pathogenic bacteria, both Gram (+) and gram (−). Among them, the most sensitive bacteria were *Listeria monocytogenes* and *Enterococcus faecalis* (average inhibition zones of 20.35 and 19.71 mm, respectively), and *S. typhimurium* and *S. aureus* showed moderate resistance [112].

Some studies compared the antimicrobial activity between different species of wild fruits. In a study, polyphenolic extracts of three wild red berry fruits, European cornel (*Cornus mas*), blackthorn (*Prunus spinosa*), and blackberry (*Rubus fruticosus*), were assessed for their antimicrobial activities by the disc diffusion method. Almost all the tested bacterial strains (such as *E. coli*, *P. aeruginosa*, and *Salmonella enteritidis*) were inhibited by all extracts. *S. enteritidis* was the most sensitive among Gram (−) bacteria, while *S. aureus* was the most sensitive among Gram (+) bacteria. Blackthorn extract showed slightly higher antimicrobial activity compared with the other tested extracts [96]. In addition, Turker et al. tested the antimicrobial activity of eight wild fruits grown in Turkey [113]. Results showed that fresh fruits of wayfaring tree, firethorn, and hawthorn showed the highest antibacterial activity. In addition, ethanol extracts of these fruits exhibited strong inhibitory effects on *S. aureus*, *Staphylococcus siepidermidis*, and *Streptococcus pyogenes* [113]. Furthermore, fruits of three wild plants growing in Mexico, namely nanchi (*Byrsonima crassifolia*), arrayan (*Psidium sartorianum*), and ayale (*Crescentia alata*), were analyzed by Pio-Leon et al. They not only measured their antibacterial activities against 21 human pathogenic bacteria by the micro-dilution assay, but also established the minimum inhibitory concentration (MIC) and minimum bactericide concentration (MBC). Results showed that methanol extracts of arrayan exhibited the highest activity against the Gram (+) bacteria, being most sensitive to *S. aureus*. Meanwhile, hexane extracts of arrayan and ayale exhibited the highest inhibitory effects on enterobacteria (*E. coli*, *Salmonella* spp., and *Shigella* spp.) [114]. Moreover, it was found that essential oils isolated from the fruits of wild *Hypericum perforatum* and *Hypericum scabrum* exhibited higher antimicrobial activity against *S. aureus* and *E. coli* than their main constituent, α-pinene [41]. Additionally, methanol and hexane extracts from a pulp of wild tamarind fruit (*Tamarindus indica*) were tested for their inhibitory activities on human pathogenic microorganisms including five bacteria and three fungi. All the bacterial strains showed sensitivity to both extracts, while only *Penicillium* species were sensitive to hexane extract [115]. In addition, several water and methanol extracts of the 16 cultivars selected from Taiwanese indigenous wild bitter gourd (*Momordica charantia*) showed inhibitory activity against the growth of *E. coli* and *Salmonella enterica* [116]. Results of another study revealed that a petroleum ether extract of wild *Atriplex inflata* fruits possessed high inhibitory activity against *Botrytis cinerea* [117].

#### 2.2.2. Antiviral Activity

Several wild fruits have exhibited antiviral activity. Knox et al. detected antiviral properties of crude extracts of wild Kurokarin (*Ribes nigrum*) fruit against influenza virus types A and B (VIA and VIB). At a concentration of 3.2 μg/mL, plaque formation of both IVA and IVB was inhibited by the extract by 50% (IC_50_). Additionally, when treating the host cells with 10 and 100 μg/mL of the extract for 6 h after infection, the growth of IVA could be completely suppressed. Virus titers in culture fluids of the cells were completely suppressed after treatment with 100 μg/mL of Kurokarin extract for 1 h after infection of 8 to 9 h, indicating that the extract inhibited the virus release from the infected cells [118]. Furthermore, extracts of a series of wild berry fruit from Bulgaria possessed great antiviral activities [119]. Four wild berries, strawberry, raspberry, bilberry, and lingonberry, were tested for their antiviral properties against some important human pathogens, poliovirus type 1 (PV-1), coxsackievirus B1 (CV-B1), human respiratory syncytial virus A2 (HRSV-A2), and influenza virus (A/H3N2), by virus cytopathic effect inhibition test. It was revealed that extracts of all berry fruits suppressed proliferation of CV-B1 and influenza virus A/H3N2. Meanwhile, anthocyanin fractions of all wild berries showed a considerable inhibitory effect against the replication of influenza virus A/H3N2.

These studies proved that wild fruits could function as potent antibacterial, antifungal, and antiviral agents. The antimicrobial activities of some wild fruits are summarized in Table 2.

### 2.3. Anti-Inflammatory Activity

Inflammation is closely related to various diseases, such as atherosclerosis, heart disease, stroke, cancer, diabetes mellitus, bone arthritis, asthma, migraine pain, periodontitis, irritable bowel syndrome, and chronic fatigue syndrome. Currently, drugs used to treat chronic inflammatory diseases are mainly various nonsteroidal drugs, which may exert side effects [120]. Therefore, the development of effective and natural sources of anti-inflammatory products has gained increasing attention. Evidence accumulated in recent years pointed out that several kinds of wild fruits possess anti-inflammatory activities, through various mechanisms of action.

Nitric oxide (NO) is a marker of late inflammation formed during activation of inducible nitric oxide synthase (iNOS) [121], and chemokine (C–C motif) ligand 20 (CCL20) is an important chemokine for immune and inflammatory response [122]. Therefore, the inhibition of NO and CCL20 is an indicator of possible anti-inflammatory properties. In the research conducted by Fazio et al., in vitro anti-inflammatory activities of the methanol extracts from the seeds of wild blackberry (*Rubus ulmifolius*) and elderberry (*Sambucus nigra*) were analyzed [75]. They firstly evaluated the seeds’ ability to inhibit lipopolysaccharide (LPS) induced NO production in mouse macrophage cell line RAW264.7 macrophages. Results showed that wild blackberry extract decreased NO release with almost 60% inhibition at the highest dose (50 μg/mL). Meanwhile, it showed a concentration-dependent effect. Subsequently, the influence of both extracts on macrophage-inflammatory protein-3α/CCL20 were evaluated. Wild blackberry extract decreased CCL20 production in a concentration-dependent manner, with a more than 90% inhibition at 50 μg/mL. By comparison, wild elderberry extract did not show a significant effect on decreasing either NO or CCL20 production. The results confirmed that wild blackberry possessed a strong anti-inflammatory activity.

Metabolites of the 5-lipoxygenase (5-LOX) pathway are important mediators of inflammation. LOX and its metabolites are shown to play a vital part in tumor formation and cancer metastasis. In some cancer cells, such as prostate, lung, colon, and breast, high expression of 5-LOX was found. In one study, the anti-inflammatory activity of *Ziziphus mistol* ripe berries, an exotic Argentinean fruit, was tested. The three tested extracts (ethanolic mistol extraction, aqueous mistol extraction, and acetone water mistol extract) were obtained after two different processes: boiling and hydroalcoholic extraction. They determined LOX activity to evaluate anti-inflammatory activity. In working conditions, only an ethanolic extract exhibited inhibition of LOX activity (IC_50_ = 183.80 μg gallic acid equivalents (GAE)/mL), while an aqueous extract showed no inhibitory effect at the tested concentrations (until 45.08 μg GAE/mL). These results suggested that bioactive compounds might be thermolabile, yet *Ziziphus mistol* ripe berries still had potent anti-inflammatory activity [30].

Cyclooxygenase-2 (COX-2) expression is an important pro-inflammatory response. Several studies have confirmed that COX-2, an important inflammatory mediator, is closely related to the occurrence and development of diabetes mellitus and diabetic nephropathy. Therefore, the inhibition of COX-2 is an indicator of possible anti-inflammatory properties. A study analyzed the anti-inflammatory activity of three wild Jamaica-grown fruits species (*Rubus jamaicensis*, *Rubus rosifolius*, and *Rubus racemosus*) and three wild Michigan-grown species (*Rubus acuminatus*, *Rubus idaeus* cv., and *Rubus idaeus* cv.). The COX-1 and COX-2 enzyme inhibitory activities were measured by monitoring the initial rate of O_2_ uptake. Aspirin, Celebrex, and Vioxx were used as positive controls. Results showed that all the hexane extracts of the Jamaica-grown *Rubus* berries were COX-active, inhibiting COX-2 by 18%–33%, while the Michigan-grown *Rubus* extracts were, in general, not COX-active [123]. In another study, eight compounds separated from the ethyl acetate extract of the *Rubus rosifolius* growing wild in elevated regions in Jamaica were identified as euscaphic acid, 1-b-hydroxyeuscaphic acid, hyptatic acid B, 19α-hydroxyasiatic acid, trachelosperogenin, 4-epi-nigaichigoside F1, nigaichigoside F1, and trachelosperoside B-1 by nuclear magnetic resonance (NMR) spectroscopy. In vitro COX-1 and COX-2 enzyme inhibitory assays were conducted to evaluate anti-inflammatory activity. Euscaphic acid, 1-b-hydroxyeuscaphic acid, and hyptatic acid B showed selective COX-1 enzyme inhibitory activity (13%, 25%, and 35% respectively) at 25 μg/mL. Similar COX inhibitory activity was demonstrated by compounds 4-epi-nigaichigoside F1 and trachelosperoside B-1, which showed moderate selectivity against the COX-1 enzyme [124]. In addition, the anti-inflammatory activity of *Psidium cattleianum* (strawberry guava) was analyzed using COX-1 and -2 enzyme inhibitory assays. Results showed that ethyl acetate extract of guava exhibited notable activity (56.4%) against the COX-2 isoform, followed by methanolic extract (44.1%) against the COX-1 enzyme at 250 μg/mL [111]. Furthermore, a polyphenol-rich fraction from lowbush cranberry, a wild Alaskan *Vaccinium* berry, showed effective inhibition of LPS-elicited induction of interleukin-1β (IL-1β) in RAW 264.7 cells [125]. Some wild fruits are rich in anthocyanins, which are known to possess antioxidant and anti-inflammatory activities. A study evaluated the inhibitory effects of wild blackberries on pro-inflammatory responses (NO production, iNOS expression, COX-2 expression, and prostaglandin E2 level). Results demonstrated that dietary consumption of wild blackberries (*Rubus* spp.) could decrease NO-generated oxidative stress and inhibit the expression of pro-inflammatory proteins, thus protecting the body against oxidation- or inflammation-related diseases [120]. The macrophage cell line RAW 264.7 was stimulated by LPS to cause pro-inflammatory responses. Different fractions from wild blackberry genotypes (WB-3, WB-7, WB-10, and WB-11) were tested separately. At 50 μM (cyanidin-3-*O*-glucoside or catechin equivalent), all markers were significantly (*p* < 0.05) inhibited by most fractions. The highest NO inhibition was observed in the anthocyanin-rich fraction from WB-10, the highest inhibitory activity on iNOS expression was presented by proanthocyanidin-rich fractions from the WB-10, and polyphenolic-rich fractions from WB-7 were identified as potent inhibitors of COX-2 expression.

Nuclear factor-κB (NF-κB) plays an important part in immune, stress, inflammatory, proliferative, and apoptotic responses [126]. The inhibition of NF-κB is commonly considered as an effective strategy to treat inflammatory disorders [127]. Tumor necrosis factor-α (TNF-α) and interleukin (IL) are important inflammatory cytokines. In a study, the anti-inflammatory activities of wild lowbush blueberry were investigated. Effects of the phenolic acid (PA) mixture were firstly measured by the inhibition against LPS-induced NF-κB activation, and results showed that NF-κB activation was significantly inhibited (by 33.2% at 4 mg FBE/mL) by PA mixture. Based on the result, a concentration of 4 mg FBE/mL was used in TNF-α and IL-6 ELISA. The production of both TNF-α (36.7%) and IL-6 (37.5%) were significantly decreased by the PA mixture. In conclusion, a phenolic acid mixture of lowbush blueberry showed anti-inflammatory activities by inhibiting NF-κB activation and the production of inflammatory cytokines (TNF-α and IL-6) at a high dose [128]. In addition, Hsu et al. did a relatively comprehensive experiment on the anti-inflammatory property of wild bitter melon (WBM), including both in vitro and in vivo experiments [129]. Inflammation was induced by *Propionibacterium acnes*. Results showed that in vitro, an ethyl acetate (EA) extract of WBM fruit potently suppressed pro-inflammatory cytokine (IL-8, TNF-α, and IL-1β) and matrix metalloproteinase (MMP)-9 levels in *P. acnes*-stimulated THP-1 cells. As for in vivo, *P. acnes*-induced ear swelling and granulomatous inflammation in mice were effectively attenuated by concomitant intradermal injection of EA extract. This study indicated that wild bitter melon could produce an anti-inflammatory effect. 

Several subfractions of *Aristotelia chilensis* have shown a notable inhibition on the 12-deoxyphorbol-13-decanoate (TPA)- induced inflammation in ear of the mouse edema (EC_50_ of 0.3 to 11.8 μg/mL) [130]. In another study conducted by the same researchers, results showed that carrageenan-induced inflammation in the rat paw was inhibited by these samples [131]. Similarly, in the inflammatory pain mice models induced by acetic acid and formalin, abdominal constrictions and the inflammatory phase of nociception were significantly reduced by intraperitoneal administration of a fraction separated from tamarillo (*Solanum betaceum*), a tropical exotic fruit. The results suggested that the fraction had a possible antinociceptive effect on inflammatory pain models [132].

These studies strongly proved that some wild fruits could be good natural sources of anti-inflammatory materials through different mechanisms of action, such as inhibiting COX-2 and NF-κB, as well as decreasing NO and CCL20 release. The anti-inflammatory activities of some wild fruits are summarized in Table 3.

### 2.4. Anticancer Activity

Cancer is known as a major cause of death all over the world. A relationship between fruit intake and a reduced risk of cancer has been found [133,134]. Various natural products, such as fruits, vegetables, and herbal plants, have been widely proved to possess antiproliferative activities [135,136,137]. Several wild fruits, such as wild red raspberry from Jamaica, and wild blueberry, have been proven to possess anticancer activities against breast, colon, prostate, and cervical cancer cells.

Malta et al. tested the inhibitory activity on tumor cell proliferation of three kinds of exotic Brazilian fruits, gabiroba (*Campomanesia cambessedeana*), murici (*Byrsonoma verbascifolia*), and guapeva (*Pouteria guardneriana*), by the MTS assay [77]. The gabiroba, murici, and the pulp of guapeva inhibited growth of HepG2 cell in a dose-dependent manner, with EC_50_ values of 40.7 ± 4.8, 173.6 ± 18.2, and 37.9 ± 2.2 mg/mL, respectively. The extracts were nontoxic at the concentrations used in the experiments. In another study, hyptatic acid B and 4-epi-nigaichigoside F1 compounds separated from ethyl acetate extract of wild *Rubus* fruits inhibited the growth of human colon tumor cells by 56% and 40%, respectively [124]. In another study, eight different extracts of each wild fruit were tested for anticancer activity. Results showed that the greatest anticancer activity was obtained from a cold water extract of fresh *R. caesius* fruit (100% inhibition), followed by cold and hot ethanol extracts of fresh *V. lantana* fruit (90.5% and 95.2% inhibition, respectively) [114]. 

Proanthocyanidin is a general term for a large class of polyphenols, which is composed of catechin, epicatechin, and epicatechin gallate in forms of different degrees of polymerization (DPn). Some studies have proved that proanthocyanidin possessed various kinds of bioactivities, such as antioxidant and anticancer activities, preventing hepatic and brain lipid peroxidation and DNA damage in animals [138]. The antiproliferative activity of proanthocyanidin-rich extracts from wild blueberry (*Vaccinium angustifolium*) was tested. Results showed that the antiproliferative activity of different fractions was positively correlated with proanthocyanidin content, and the fraction with a DPn of 5.65 showed considerable antiproliferative activity against human prostate and mouse liver cancer cell lines [139]. Results also suggested that antiproliferative activity was associated with high molecular weight proanthocyanidin oligomers from wild blueberry fruits.

Yellow Himalayan raspberry, as a wild edible fruit, was analyzed for antiproliferative activities. Results showed that acetone and methanol extracts exhibited inhibitory effects against human cervical cancer cells (C33A) (EC_50_ at 5.04 and 4.9 mg/mL fruit concentration respectively), and were nontoxic to normal peripheral blood mononuclear cells at the same time [140]. In addition, three wild species of strawberries (*Fragaria virginiana*, *Fragaria Chiloensis*, and *Fragaria xananassa*) were analyzed for antiproliferative activity. Extracts of the three fruits all significantly inhibited the proliferation of A549 human lung epithelial cancer cells [141]. In another study, Woguem et al. found that the volatile oil from the wild pepper could inhibit the growth of human tumor cells MDA-MB 231 (breast adenocarcinoma), A375 (malignant melanoma), and HCT116 (colon carcinoma), in a concentration-dependent manner [59]. Several kinds of water and methanol extracts of wild bitter gourd also showed similar cytotoxic activities on human fibrosarcoma HT 1080 cells to 10 μg/mL of doxorubicin, which was used as positive control in this study [103]. Finally, the anticancer activities of several wild fruits are summarized in Table 4.

### 2.5. Other Bioactivities of Wild Fruits

In addition to the biological activities mentioned above, some wild fruits have shown other beneficial health effects.

Some wild fruits have shown anti-acetylcholinesterase activity. The acetylcholinesterase inhibitory activity is a commonly used pharmacological model of Alzheimer’s disease. In one study, a water extract of *Sorbus torminalis* (wild service tree) fruit showed moderate ability to inhibit acetylcholinesterase [29]. Similarly, three exotic fruits from Brazil were tested for anti-acetylcholinesterase activities, namely genipap (*Genipa americana*), umbu (*Spondia tuberosa*) and siriguela (*Spondia purpurea*). Results showed that ethanol extracts of genipap pulp and siriguela seed could present a similar inhibitory effect on acetylcholinesterase compared with carbachol (positive control) [77]. In another study, an obvious cognitive enhancement was observed in the experimental mice after short-term intraperitoneal supplementation with a polyphenol-rich extract of wild blueberries (*Vaccinium angustifolium*) [98]. Researchers found that the brain antioxidant properties of mice were higher and acetylcholinesterase activity was inhibited after the treatment, indicating that bioactive components of wild blueberry are able to affect the brain function of mice in a positive way.

Furthermore, larvicidal/insecticidal activities have been observed in several wild fruits. The researchers evaluated the insecticidal activity of wild *Tetradium glabrifolium* fruits against *Aedes albopictus* [142]. Essential oils and three compounds from the fruit showed strong larvicidal activities against the early fourth-instar larvae of *A. albopictus*. In another study, the antigiardial activities of wild watermelon (*Citrullus lanatus*) fruits were investigated [143]. Results revealed that two compounds from the fruits, cucurbitacin L 2-*O*-β-glucoside and cucurbitacin E, had potent antigiardial activity against *Giardia lamblia* in vitro. Meanwhile, all the extracts, including petroleum ether, ethyl acetate, and butanol crude extracts, were active against *Giardia lamblia*. The results indicated that this fruit might be a potential new resource for the control of giardiasis. *Zanthoxylum schinifolium* is a traditional wild Chinese medicinal plant. Researchers found that essential oils of the fruits exhibited strong fumigant toxicity against the maize weevil *Sitophilus zeamais*, a common grain storage insect [144]. Similarly, fruits of another wild Chinese medicinal plant called *Carum carvi* showed strong fumigant toxicity and contact toxicity against *Sitophilus zeamais* and *Tribolium castaneum* adults, which are both common grain storage insects [145].

There was a study proving that 70% methanol extract of *Elaeagnus latifolia*, a wild edible fruit, had a promising effect on protecting pUC18 DNA [37]. In addition, a methanol extract of wild *Brenania brieyi* fruit showed estrogenic effects by doubling the uterine weight and increasing the vaginal epithelial height of female rats [146]. Advanced glycation endproducts (AGE) is an important related pathophysiological feature common to many chronic diseases, such as cardio- and cerebrovascular diseases, diabetes mellitus, and Alzheimer’s disease. Inhibitory activity on AGE formation was related to radical scavenging activities. In a study, all samples of wild berries reduced AGE formation in a concentration-dependent way, with a positive correlation to each extract’s total phenolic content and, to a lesser degree, total anthocyanin content [147]. Moreover, it has been reported that methanol extracts of wild raspberry fruits had potassium-conservation diuretic activity in experimental rats [148]. The fruit of wild *Aristotelia chilensis* also showed gastroprotective effects and thus have great potential as nutraceuticals [131].

**Figure 1:**
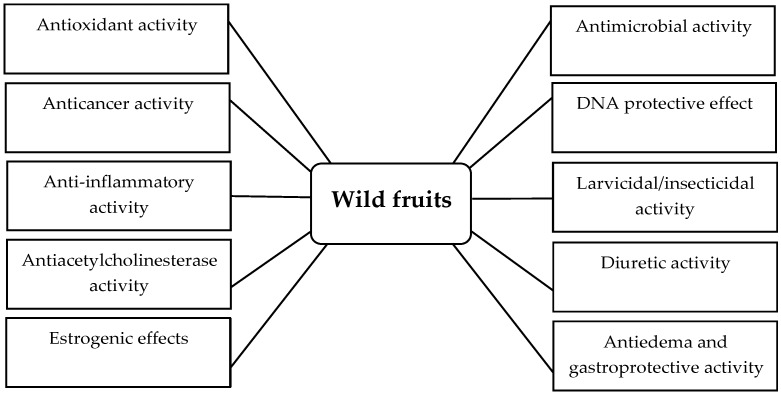
Some bioactivities of wild fruits.

Other bioactivities of wild fruits are summarized in Table 5. All the bioactivities of wild fruits are displayed in Figure 1.

## 3. Bioactivities of Wild Berries

The berries are an important group of fruits. Berries include members of several families, such as *Rosaceae* and *Ericaceae* [149]. It is well established that berries contain high contents of bioactive compounds, such as phenolic acids, anthocyanins, flavonols, and tannins [150,151]. Wild berries are so far underutilized, but they are often equal to or more valuable than commercial berries in terms of their bioactivities and health benefits, such as antioxidant, antimicrobial, anti-inflammatory, and anticancer activities [78,104,123,139]. The bioactivities of wild berries involved in this review are summarized in Table 6.

## 4. Conclusions

The special genotype and formative environment create unique and abundant ingredients with health benefits in wild fruits. When a wild species is domesticated, the biological activities might decrease. In addition, wild fruits should not be excessively exploited, as this could cause a depauperation of the natural environment. Various kinds of wild fruits have shown numerous bioactivities, such as antioxidant, antimicrobial, anti-inflammatory, anticancer, and anti-acetylcholinesterase activities. Some wild fruits have more than one bioactivity. For example, *Aristotelia chilensis* possesses anti-inflammatory, antiedema, and gastroprotective activities. The consumption and utilization of some wild fruits have been increasing, and some wild fruits have been developed into functional foods. In the future, for full utilization of wild fruit resources, more bioactivities of wild fruits should be evaluated, and bioactive components should be isolated and identified. The mechanisms of action should be explored further. In addition, the toxicological evaluation of some wild fruits is also necessary for safe human consumption.

## Figures and Tables

**Table 1:** Antioxidant activities of some wild fruits.

Wild Fruits	Bioactive Compounds	Effects	References
56 wild fruits from South China	polyphenols	antioxidant activity	[3]
*Prunus mahaleb*	total anthocyanin and phenolics	scavenging free radicals (oxygen radicals)	[27]
*Psidium acutangulum*	phenolics, citric, annurcoic, ω3, ω6, ω9 fatty acids, and ascorbic acid	scavenging free radicals (DPPH, ABTS)	[28]
*Sorbus torminalis*	phenolic compounds	scavenging free radicals (ABTS, superoxide anion radicals), antioxidant activity	[29]
*Ziziphus mistol*	polyphenols	scavenging free radicals (ABTS, DPPH, superoxide and hydroxyl radicals)	[30]
*Annona cherimola*	not mentioned	scavenging free radicals (DPPH, ABTS), antioxidant activity, inhibition of lipid peroxidation	[31]
*Myrica esculenta*	polyphenols	scavenging free radicals (DPPH, ABTS), antioxidant activity	[32]
*Vaccinium meridionale*	phenolic compounds	scavenging free radicals (ABTS), antioxidant activity	[33]
*Lycium ruthenicum*	polyphenols	scavenging free radicals (DPPH, ABTS), antioxidant activity	[34]
*Malpighia ernarginata*	phenolic acids	scavenging free radicals (DPPH, ABTS, oxygen radical)	[35]
*Morus rubra*	not mentioned	antioxidant activity	[36]
*Bunium persicum*	Phenolics and flavonoids	scavenging free radicals (DPPH), antioxidant activity	[37]
*Elaeagnus latifolia*	phenolics and flavonoids	scavenging hydroxyl radicals, superoxide radicals, singlet oxygen radicals, hypochlorous acid	[38]
*Solanum incanum*	3-*O*-acetyl-and4-*O*-acetyl-5-*O*-(*E*)-caffeoylquinic acids	scavenging free radicals (ABTS, DPPH) and iron chelation activity	[39]
*Rosa canina*	α-tocopherol, β-carotene, reducing sugar, and ascorbic acid	scavenging free radicals (DPPH), reducing power, inhibition of β-carotene bleaching and lipid peroxidation	[40]
*Mespilus germanica*	not mentioned	scavenging nitric oxide and H_2_O_2_ radicals, inhibition of lipid peroxidation	[41]
*Aristotelia chilensis*	phenolics	scavenging free radicals (DPPH, superoxide radicals, oxygen radicals), antioxidant activity, inhibition of lipid peroxidation	[42]
*Myrtus communis*	not mentioned	scavenging free radicals (DPPH, β-carotene-linoleic acid)	[43]
*Rubus hirsutus*	Phenolics and flavonoids	scavenging free radicals (DPPH), antioxidant activity	[44]
*Piper capense*	not mentioned	scavenging free radicals (ABTS)	[45]
*Vitis coignetiae*	anthocyanins	scavenging free radicals (ABTS, DPPH)	[46]
*Syzygium cumini*	phenolics, tannins, and anthocyanins	scavenging free radicals (DPPH, hydroxyl radical and superoxide radical), inhibition of lipid peroxidation	[48]
*Vatis amurensis*	catechin, epicatechin, 4-methyl-catechol, gallic, protocatechuic, chlorogenic, caffeic, p-coumaric, and syringic acids	scavenging superoxide radicals	[49]
14 wild genotypes of *Citrus reticulata*	not mentioned	scavenging free radicals (DPPH, ABTS, oxygen radicals), antioxidant activity	[50]
10 crabapples (*Malus* wild species)	polyphenols, flavonoids	scavenging free radicals (DPPH, ABTS), antioxidant activity	[51]
*C. speciosa*, *C. thibetica*, *C. cathayensis*, *C. sinensis*, *C. japonica*	polyphenols	scavenging free radicals (DPPH, ABTS), antioxidant activity	[52]
wild genotype of *Vaccinium* spp.	anthocyanin, polyphenols	scavenging free radicals (ABTS, superoxide anion and hydroxyl radical)	[53]
*Hypericum perforatum*, *Hypericum scabrum*	α-pinene	scavenging free radicals (DPPH), inhibition of β-carotene bleaching	[54]
wild *Fragaria* genotypes	not mentioned	antioxidant activity	[55]
*Ensete superbum*	Phenolics and tannin	scavenging free radicals (DPPH, ABTS), antioxidant activity	[56]
*Fragaria vesca*	phenolics	scavenging free radicals (DPPH), antioxidant activity	[57]
*Fragaria vesca*	not mentioned	scavenging free radicals (DPPH)	[58]
wild strawberries	not mentioned	antioxidant activity	[59]
2 wild raspberries	not mentioned	scavenging free radicals (DPPH, ABTS), antioxidant activity	[60]
6 genotypes of *Diospyros kaki*	gallic acid, vanillic acid, caffeic acid, syringic acid, and quercetin	scavenging free radicals (DPPH, ABTS, hydroxyl radical), antioxidant activity	[61]
*Rosa canina*	polyphenols and vitamin C	scavenging free radicals (DPPH)	[62]
*Momordica charantia*	not mentioned	scavenging free radicals (DPPH, hydroxyl radicals), protection against Cu^2+^-induced low-density-lipoprotein peroxidation	[63]
*Prunus amygdalus*	not mentioned	scavenging free radicals (DPPH), reducing power	[64]
2 wild blueberries	polyphenols	scavenging free radicals (DPPH, ABTS, oxygen radicals), antioxidant activity	[65]
*Citrus hystrix*	phenolics	scavenging free radicals (DPPH), antioxidant activity	[66]
*Amygdalus lycioides*, *Amygdalus kotschyi*, *Amygdalus pabotti*, *Amygdalus trichamygdalus*	phenolics	scavenging free radicals (nitrite, hydrogen peroxide, superoxide radicals), reducing power	[67]
*Vaccinium miyrtillus*	phenolics	scavenging free radicals (DPPH), antioxidant activity	[68]
*Rubus croceacanthus* and *Rubus sieboldii*	anthocyanins, ascorbic acid	scavenging oxygen radicals	[69]
wild cranberry	not mentioned	scavenging free radicals (ABTS)	[70]
wild blueberry and cranberry	not mentioned	scavenging free radicals (DPPH)	[71]
*Ugni molinae*	polyphenols	scavenging free radicals (DPPH, ABTS)	[72]
12 native Australian fruits	total phenolics	scavenging free radicals	[73]
14 species of wild fruits	phenolics and flavonoids	scavenging free radicals (DPPH, ABTS), antioxidant activity	[74]
*Rubus ulmifolius* and *Sambucus nigra*	phenolics	scavenging free radicals (DPPH)	[75]
*Campomanesia cambessedeana*, *Byrsonoma verbascifolia*, *Pouteria guardneriana*	phenolics and flavonoids	scavenging free radicals (oxygen radicals, peroxyl radicals), cellular antioxidant activity	[76]
*Genipa americana*, *Spondia tuberose*, *Spondia purpurea*	chlorogenic acid	scavenging free radicals (ABTS), antioxidant activity, inhibition of lipid peroxidation in a biomimetic membrane system and mouse liver, inhibition of lipid peroxidation in mouse liver	[77]
*Rubus megalococcus, Myrciaria aft cauliflora, Hyeronima macrocarpa*	anthocyanin	scavenging free radicals (ABTS, DPPH)	[78]
*11 exotic fruits from Brazil*	phenolics	scavenging free radicals (DPPH, ABTS)	[79]
*15 wild fruits*	polyphenols	scavenging free radicals (DPPH)	[80]
*Ximenia caffra*, *Sclerocarya birrea*, *Parinari curatellifolia*, *Vitex payos*, *Bridelia molis*, *Berchemia zeyheri*	not mentioned	scavenging free radicals (DPPH, superoxide anion radical), reducing power, inhibition of phospholipids peroxidation	[81]
cambuci, araca-boi, camu-camu, jaracatia, araca	not mentioned	scavenging free radicals (DPPH)	[82]
23 wild blueberry fruits	phenolic compounds	scavenging free radicals (ABTS), antioxidant activity	[83]
*Garcinia pedunculata*, *Garcinia xanthochymus*, *Docynia indica*, *Rhus semialata* and *Averrhoa carambola*	phenolics	antioxidant activity	[84]
*Arbutus unedo*, *Rubus ulmifolius*	phenolic acids, anthocyanins, ascorbic acid	scavenging free radicals (ABTS, DPPH), antioxidant activity	[85]
*Prunus spinosa* and *Crataegus monogyna*	phenolic compounds	scavenging free radicals (DPPH, ABTS), antioxidant activity	[86]
wild bacuri, caja, camu-camu, carnauba, gurguri, jabuticaba, jambolao, jucara, murta, black puca and puca fruits	not mentioned	scavenging free radicals (DPPH)	[87]
*Crataegus azarolus*, *Crataegus monogyna*, *Prunus spinosa*, *Rosa canina*, *Rubus ulmifolius*, *Sorbus domestica*	Phenolics and carotenoids	scavenging free radicals (ABTS, H_2_O_2_)	[88]
*Diospyros mespiliformis*, *Flacourtia indica*, *Uapaca kirkiana* and *Ziziphus mauritiana*	not mentioned	scavenging free radicals (DPPH, superoxide anion radical), reducing power	[89]
*Fragaria indica*, *Prunus armeniaca*, *Pyracantha crenulata* and *Rubus ellipticus*	not mentioned	scavenging free radicals (DPPH, ABTS), antioxidant activity	[90]
20 exotic fruits	not mentioned	scavenging free radicals (DPPH, ABTS, oxygen radicals), antioxidant activity	[91]
24 exotic Colombian fruits	soluble phenolics	scavenging free radicals (ABTS), antioxidant activity	[92]
wild abiu, acerola, wax jambu, cashew, mamey sapote, carambola or star fruit, Surinam cherry, longan, sapodilla and jaboticaba fruits	not mentioned	scavenging free radicals (hypochlorous acid, ABTS, and DPPH)	[93]
exotic araca-boi, cajamanga, sirihuela, dovialis, landim, murici, tomatinho do mato fruits	phenolics	scavenging free radicals (ABTS, DPPH), antioxidant activity	[94]
17 exotic fruits	phenolics and proanthocyanidins	antioxidant activity	[95]
*Cornus mas*, *Prunus spinosa*, *Rubus fruticosus*	polyphenolics	scavenging free radicals	[96]
*Salacca edulis Reinw*, *Garcinia mangostana*	phenolics	hindering the rise in plasma lipids and decrease of antioxidant activity in rats fed with cholesterol	[97]
*Vaccinium angustifolium*	polyphenols	improving brain antioxidant properties in mice (antioxidant activity, improving ascorbic acid concentration and glutathione levels, reducing lipid peroxidation products)	[98]
wild durian, snake fruit and mangosteen	not mentioned	scavenging free radicals (ABTS, DPPH), antioxidant activity	[99]

ABTS: 2,2′-azinobis-3-ethylbenzothiazoline-6-sulphonate; DPPH: 2,2-diphenyl-1-picrylhydrazyl.

**Table 2:** Antimicrobial activities of some wild fruits.

Wild Fruits	Bioactive Compounds	Effects	References
*Hypericum perforatum*, *Hypericum scabrum*	not mentioned	inhibition of *S. aureus* and *E. coli*	[54]
*Cornus mas*, *Prunus spinosa*, *Rubus fruticosus*	polyphenols	inhibition of all the tested bacterial strains	[96]
*Piper capense*	not mentioned	inhibition of *S. aureus*, *E. faecalis*, and *C. albicans*	[45]
*Nitraria retusa*	not mentioned	inhibition of *S*. *typhimurium*, *K. pneumonia*, and *B. thuringiensis*	[102]
*Crataegus azarolus*	phenolics	inhibition of *S. aureus* and *S. faecalis*	[103]
*Prunus mahaleb*	not mentioned	inhibition of some Gram (+) and Gram (−) bacteria and fungi	[104]
*Clematis apiifolia*	protoanemonin	inhibition of various yeasts and non-lactic acid bacteria	[105]
*Momordica charantia*	not mentioned	inhibition of *Mycobacterium tuberculosis*	[106]
*Scabiosa arenaria*	not mentioned	inhibition of some bacteria, *Candida* species, and phytopathogenic fungi	[107]
*Carissa opaca*	not mentioned	inhibition of some bacteria	[108]
*Olea ferruginea*	not mentioned	inhibition of some Gram (+) and Gram (−) bacteria	[109]
*Citrullus lanatus*	not mentioned	inhibition of *S. aureus*, *B. subtilis*, *P. valgaris*, and *P. aerguinosa*	[110]
*Psidium cattleianum*	not mentioned	inhibition of *B. subtilis* and *S. aureus*	[111]
*Ribes nigrum* L.	not mentioned	inhibition influenza virus types A and B	[118]
*Viburnum lantana*, *Pyracantha coccinea*, *Crataegus monogyna*	not mentioned	inhibition of *S. aureus*, *S. epidermidis*, and *S. pyogenes*	[113]
*Byrsonima crassifolia*, *Psidium sartorianum*, *Crescentia alata*	not mentioned	inhibition of *E. coli*, *Salmonella* spp., *Shigella* spp., and *S. aureus*	[114]
*Tamarindus indica*	not mentioned	inhibition of some human pathogenic microorganisms	[115]
*Momordica charantia*	not mentioned	inhibition of *E. coli* and *Salmonella enterica*	[116]
*Atriplex inflata*	not mentioned	inhibition of *Botrytis cinerea*	[117]
*Fragaria vesca*, *Rubus idaeus*, *Vaccinium myrtillis*, *Vaccinium vitis-idaea*	anthocyanins	inhibition of the replication of coxsackie virus B1 and influenza virus A/H3N2	[119]
wild European cranberry	not mentioned	inhibition of *E. coli* and *S. typhimurium*, *E. faecalis*, *Listeria monocytogenes*, *S. aureus*, and *B. subtilis*	[112]

**Table 3:** Anti-inflammatory activities of some wild fruits.

Wild Fruits	Bioactive Compounds	Effects	References
*Ziziphus mistol*	not mentioned	inhibition of LOX activity	[30]
*Rubus ulmifolius*, *Sambucus nigra*	not mentioned	inhibition of LPS-induced inflammatory mediators (NO, CCL20)	[75]
*Psidium cattleianum*	not mentioned	inhibition expression of COX-2 enzyme	[111]
wild blueberry (*Rubus* spp.)	anthocyanin-rich, proanthocyanidin-rich, and polyphenolic-rich fraction	inhibition expression of COX-2, NO, and iNOS	[120]
*Rubus jamaicensis*, *Rubus rosifolius*, *Rubus racemosus*	not mentioned	inhibition the expression of COX-1 and COX-2 enzymes	[123]
*Rubus rosifolius*	ursolic acid analogues	inhibition expression of COX-1 enzyme	[124]
*Vaccinium vitis-idaea*, *Vaccinium uliginosum*	polyphenol-rich fraction	inhibition of LPS-elicited induction of IL-1 β in RAW 264.7 cells	[125]
*Vaccinium angustifolium*	phenolic acids	inhibiting NF-κB activation and production of inflammatory cytokines (TNF-α and IL-6)	[128]
*Momordica charantia*	phytol and lutein	suppressing pro-inflammatory cytokine and MMP-9 levels, attenuating *P. acnes*-induced ear swelling and granulomatous inflammation in mice	[129]
*Aristotelia chilensis*	not mentioned	inhibition of carrageenan-induced inflammation in ear of the mouse edema in TPA inflammation mode	[130,131]
*Solanum betaceum*	not mentioned	antinociceptive effect on inflammatory pain mice models	[132]

LOX: lipoxygenase; LPS: lipopolysaccharide; NO: nitric oxide; COX-2: cyclooxygenase-2; iNOS: inducible nitric oxide synthase; TNF: Tumor necrosis factor, NF-κB: nuclear factor-κB; MMP: matrix metalloproteinase; TPA: 12-deoxyphorbol-13-decanoate; IL-1: interleukin-1; RAW 264.7: mouse macrophage cell line.

**Table 4:** Anticancer activities of some wild fruits.

Wild Fruits	Bioactive Compounds	Effects	References
*Campomanesia cambessedeana*, *Byrsonoma verbascifolia*, *Pouteria guardneriana*	phenolic compounds	inhibiting growth of HepG2 human liver cancer cells	[77]
*Piper capense*	essential oil	inhibiting growth of human breast adenocarcinoma, malignant melanoma, and colon carcinoma cells	[45]
*Rubus caesius*, *Viburnum lantana*, *Crataegus monogyna*, *Crataegus tanacetifolia*	not mentioned	inhibition of tumor cells	[114]
*Momordica charantia*	not mentioned	cytotoxic activities on human fibrosarcoma HT 1080 cells	[117]
*Rubus rosifolius*	hyptatic acid B, 4-epi-nigaichigoside F1	inhibiting growth of colon tumor cells	[124]
*Vaccinium angustifolium*	oligomeric proanthocyanidins fraction	inhibiting growth of human prostate and mouse liver cancer cell lines	[139]
*Rubus ellipticus*	not mentioned	inhibiting growth of human cervical cancer cells (C33A)	[140]
*Fragaria virginiana*, *F. chiloensis*, *F. xananassa*	not mentioned	inhibiting growth of A549 human lung epithelial cancer cells	[141]

**Table 5:** Other bioactivities of some wild fruits.

Wild Fruits	Bioactive Compounds	Effects	References
*Sorbus torminalis*	not mentioned	antiacetylcholinesterase activity	[29]
*Genipa americana*, *Spondia tuberosa*, *Spondia purpurea*	chlorogenic acid	antiacetylcholinesterase activity	[78]
*Elaeagnus latifolia*	phenolic and flavonoid compounds	protection of pUC18 DNA	[37]
*Vaccinium angustifolium*	polyphenol-rich extract	decreasing acetylcholinesterase activity and enhancing cognition in adult mice	[98]
*Aristotelia chilensis*	aglycone and phenolic compounds	inhibition of the carrageenan- induced inflammation in the paw rat and gastroprotective activity in rats	[131]
*Tetradium glabrifolium*	2-tridecanone, 2-undecanone and d-limonene	larvicidal activity against the early fourth-instar larvae of *A. albopictus*	[142]
*Citrullus lanatus*	cucurbitacin E, cucurbitacin L 2-*O*-β-glucoside	antigiardial activities	[143]
*Zanthoxylum schinifolium*	estragole, linalool and sabinene	fumigant toxicity against *S. zeamais*	[144]
*Carum carvi*	(R)-carvone and d-limonene	contact toxicity against *S.* and *T. castaneum* adults	[145]
*Brenania brieyi*	not mentioned	estrogenic effects	[146]
12 species of wild berries	phenolics, anthocyanins	antiglycation activity	[147]
*Rubus idaeus*	not mentioned	diuretic activity	[148]

**Table 6:** Bioactivities of some wild berries.

Bioactivity	Wild Berry	Effects	References
antioxidant activity	*Rubus megalococcus*	scavenging free radical	[78]
	*Rubus ulmifolius*	scavenging free radicals (ABTS, DPPH, H_2_O_2_), antioxidant activity	[85,88]
	*Rubus hirsutus*	scavenging free radicals (DPPH), antioxidant activity	[44]
	*Rubus ellipticus*	scavenging free radicals (DPPH, ABTS), antioxidant activity	[90]
	*Rubus croceacanthus*, *Rubus sieboldii*	scavenging oxygen radicals	[69]
	*Rubus fruticosus*	scavenging free radical (DPPH)	[96]
	*Rubus caucasicus*	antioxidant activity in β-carotene-linoleic acid, DPPH free radical scavenging, and FRAP assays	[152]
	*Vaccinium meridionale*	scavenging free radical (ABTS), antioxidant activity	[33]
	wild genotype of *Vaccinium* spp.	scavenging free radicals (ABTS, superoxide anion, and hydroxyl radical)	[53]
	*Vaccinium angustifolium*	improving brain antioxidant properties in mice (antioxidant activity, improving ascorbic acid concentration, reducing glutathione levels, reducing lipid peroxidation products)	[98]
	*Vaccinium miyrtillus*	scavenging free radicals (DPPH), antioxidant activity	[68]
	*Sorbus torminalis*	scavenging free radicals (ABTS, superoxide anion radicals), antioxidant activity	[29]
	*Sambucus nigra*	scavenging free radicals (DPPH)	[75]
	*Fragaria vesca*	scavenging free radicals (DPPH), antioxidant activity	[57,58]
	*Sorbus domestica*	scavenging free radicals (ABTS, H2O2)	[88]
	*Fragaria indica*	scavenging free radicals (DPPH, ABTS), antioxidant activity	[90]
	*Vitis coignetiae*	scavenging free radicals (ABTS, DPPH)	[46]
antimicrobial activity	wild European cranberry	inhibition of *E. coli* and *S. typhimurium*, *E. faecalis*, *Listeria monocytogenes*, *S. aureus*, and *B. subtilis*	[112]
	*Rubus fruticosus*	inhibition of all the tested bacterial strains	[96]
	*Fragaria vesca*, *Rubus idaeus*, *Vaccinium myrtillis*, *Vaccinium vitis-idaea*	inhibition the replication of coxsackie virus B1 and influenza virus A/H3N2	[119]
anti-inflammatory activity	*Rubus ulmifolius*, *Sambucus nigra*	inhibition of LPS-induced inflammatory mediators (NO, CCL20)	[75]
	*Rubus jamaicensis*, *Rubus rosifolius*, *Rubus racemosus*	inhibition the expression of COX-1 and COX-2 enzymes	[123]
	*Rubus rosifolius*	inhibition expression of COX-1 enzyme	[124]
	*Vaccinium vitis-idaea*, *Vaccinium uliginosum*	inhibition of LPS-elicited induction of IL-1 β in RAW 264.7 cells	[125]
	*Vaccinium angustifolium*	inhibiting NF-κB activation and production of inflammatory cytokines (TNF-α and IL-6)	[128]
anticancer activity	*Rubus caesius*	inhibition of tumor cells	[114]
	*Rubus rosifolius*	inhibiting growth of colon tumor cells	[124]
	*Vaccinium angustifolium*	inhibiting growth of human prostate and mouse liver cancer cell lines	[139]
	*Rubus ellipticus*	inhibiting growth of human cervical cancer cells (C33A)	[140]
	*Fragaria virginiana*, *F. chiloensis*, *F. xananassa*	inhibiting growth of A549 human lung epithelial cancer cells	[141]
